# Virtual screening as a tool to discover new β-haematin inhibitors with activity against malaria parasites

**DOI:** 10.1038/s41598-020-60221-0

**Published:** 2020-02-25

**Authors:** Ana Carolina C. de Sousa, Jill M. Combrinck, Keletso Maepa, Timothy J. Egan

**Affiliations:** 1 University of Cape Town, Department of Chemistry, Rondebosch, 7701 South Africa; 2 University of Cape Town, Division of Pharmacology, Department of Medicine, Observatory, 7925 South Africa; 3 Institute of Infectious Disease and Molecular Medicine, University of Cape Town, Rondebosch, 7701 South Africa

**Keywords:** Parasitic infection, Cheminformatics, Screening

## Abstract

Malaria remains a major public health problem. With the loss of antimalarials to resistance, the malaria burden will likely continue for decades. New antimalarial scaffolds are crucial to avoid cross-resistance. Here, we present the first structure based virtual screening using the β-haematin crystal as a target for new inhibitor scaffolds by applying a docking method. The ZINC15 database was searched for compounds with high binding affinity with the surface of the β-haematin crystal using the PyRx Virtual Screening Tool. Top-ranked compounds predicted to interact with β-haematin were submitted to a second screen applying *in silico* toxicity and drug-likeness predictions using Osiris DataWarrior. Fifteen compounds were purchased for experimental testing. An NP-40 mediated β-haematin inhibition assay and parasite growth inhibition activity assay were performed. The benzoxazole moiety was found to be a promising scaffold for further development, showing intraparasitic haemozoin inhibition using a cellular haem fractionation assay causing a decrease in haemozoin in a dose dependent manner with a corresponding increase in exchangeable haem. A β-haematin inhibition hit rate of 73% was found, a large enrichment over random screening, demonstrating that virtual screening can be a useful and cost-effective approach in the search for new haemozoin inhibiting antimalarials.

## Introduction

Malaria is an infectious parasitic tropical disease caused by five species of protozoa of the genus *Plasmodium*, of which *P*. *falciparum* is the most lethal in humans. Despite extensive efforts at eradication, malaria remains a major public health problem, mainly in economically underdeveloped regions of the world^1^. According to the World Health Organisation 2017 World Malaria Report, in 2016 91 countries reported a total of 216 million cases of malaria, an increase of 5 million cases over 2015, which resulted in 445,000 reported deaths. The sub-Saharan Africa region carries 80% of the global malaria burden^1^. These data show a troubling shift in the trajectory of this disease and suggest that much more effort is required to reach the goal of malaria eradication. One such area of work is the search for safe and efficient new treatments that ensure the rapid and complete cure of the disease^1^.

Combination chemotherapy using artesunate and amodiaquine (ASAQ) is currently one of the treatments recommended by the WHO. However, drug resistance to quinoline derivatives and the appearance of artemisinin resistance suggests that this therapy may be at risk^2^. In addition, the use of amodiaquine (AQ) can cause adverse effects such as hepatotoxicity and agranulocytosis^3^. The mechanism of action of AQ, chloroquine (CQ) and other quinolines is based on inhibition of the parasite’s mechanism of haem detoxification during the erythrocytic stage within the red blood cell (RBC), where the parasite degrades host haemoglobin to amino acids, a portion of which are used by the parasite, and free haem. This free haem is then sequestered into an inert and highly insoluble crystal called haemozoin, or malaria pigment. By interfering with this process, quinoline drugs increase the concentration of free haem in the parasite cell, which kills it, possibly via increased oxidative stress^4^. Recently, an inhibition mechanism involving drug–haemozoin crystal interaction has been supported by theoretical models and experimental evidence^5–7^. Haemozoin crystallizes as long thin needles with a triclinic morphology extending along the *c*-axis, with estimated volumes and surface areas of 0.1 μm^3^ and 2 μm^2^ respectively. It exhibits dominant, slow growing [100] and [010] side faces, a less-developed [011] face and a minor [001] face that is the fastest growing^8^. The synthetic haemozoin counterpart, β-haematin, has a crystal structure identical to haemozoin and is chemically and spectroscopically identical to haemozoin isolated from malarial trophozoites^9^.

In a recent noteworthy study, Chaparro *et al*. performed a comprehensive exercise to select those targets with the highest probability of delivering successful new drugs from the current antimalarial target portfolio^10^. Parameters related to genetic, pharmacological and chemical validation, tractability, mode of action, and therapeutic profile were included to provide a quantitative score to prioritize targets. The haemoglobin degradation pathway showed one of the highest scores in the study.

Resistance to CQ and other quinolines is associated with gene mutations encoding the *Pf*CRT protein (*P*. *falciparum* chloroquine resistance transporter) within the parasite’s digestive vacuole (DV) membrane that promotes a structure specific efflux, which is not related to the therapeutic target^11^. As a result, the haemozoin formation pathway continues to be an attractive and well-suited drug target. Nonetheless, to avoid cross-resistance new antimalarial scaffolds are crucial.

High-throughput screening (HTS) is a method to identify new leads for drug discovery which allows a large chemical library to be screened *in vitro* against a specific drug target, cell or organism. Virtual screening (VS) is a computer aided method to simulate HTS that can save time and costs in the drug development process, also reducing the failure rate by prioritising compounds for further experimental investigation. For instance, structure-based virtual screening (SBVS) uses molecular docking techniques to screen large virtual libraries of available, often purchasable chemicals that are docked with a biological target of known structure. The compounds are scored based on the predicted interactions with the target and those with the top scores (hits) are selected for experimental activity assays. Virtual screening methods have been showing success in predicting new leads with good hit rates reported^12–14^.

Thus, this work aimed at identifying new β-haematin inhibitors using a SBVS approach. In this pilot study, a part of the ZINC15 database^15^ was used to search for novel compounds with high binding affinity and high chemical complementarity with the surface of the β-haematin crystal, applying molecular docking using the PyRx Virtual Screening Tool^16^. The top-ranked compounds were submitted to a second screen employing *in silico* toxicologic and drug-likeness predictions using DataWarrior^17^. Finally, fifteen compounds were purchased to perform experimental tests. These compounds were tested using a β-haematin inhibition assay and their parasite growth inhibition activity (IC_50_) as well as cytotoxicity in mammalian cells were determined.

## Results and Discussion

### Virtual screening

Docking is a molecular modelling method that allows compounds to be screened in silico before testing experimentally. Currently, it is the best alternative to rapidly predict binding conformations of ligands that are energetically favourable to interact with a pharmacological receptor site and has gained popularity as a means to save time and costs in the drug discovery and development pipeline. With the assistance of docking studies, thousands of compounds can be assessed for potential pharmacological activity at low cost and in a short time^18^. To find new β-haematin crystal growth inhibitors, we performed SBVS using the docking approach on a sample portion of the ZINC15 database. ZINC15 is a public access database and tool set, initially developed to enable ready access to compounds for virtual screening, ligand discovery, pharmacophore screening, force field development and other cheminformatics applications. Widely useful as a research tool for ligand discovery, ZINC15 connects biological activities of gene products, drugs, and natural products with commercial availability^15^. Of the structures available, the subset selected were those with clean reactivity that were in stock for purchase, with LogP <5 and molecular mass <425. The result was a final number of 7,070 compounds.

**Figure 1:**
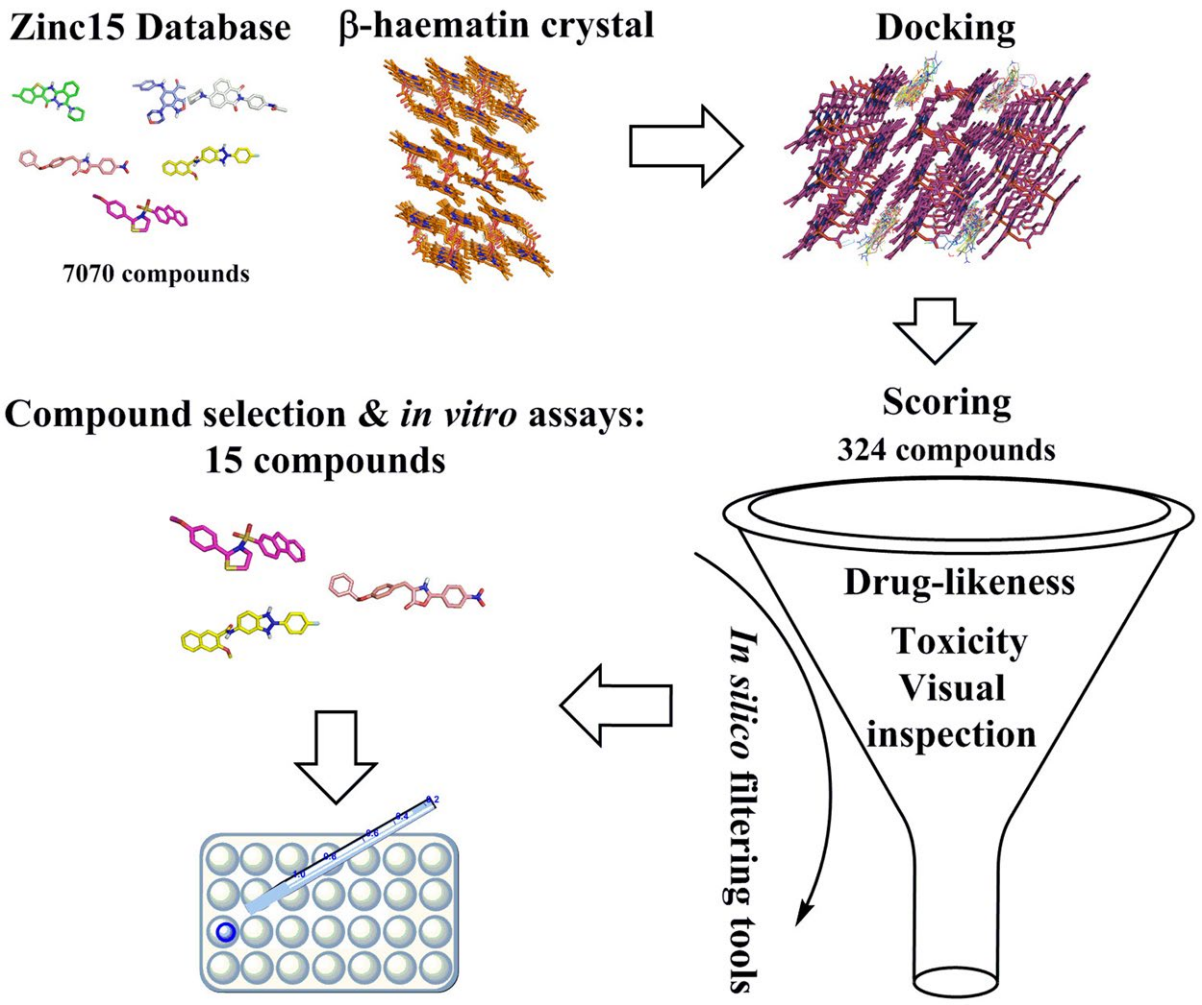
Schematic illustration of the VS protocol used in this investigation. Compounds for *in silico* docking were sourced from the ZINC15 database. Only those with suitable properties (logP < 5, MW < 425, reactivity clean and in stock) were considered. These were docked with a β-haematin crystal using the PyRx virtual screening tool. The top 324 hits were further filtered for drug-like properties using OSIRIS DataWarrior and 15 compounds purchased for experimental testing.

Following energy minimization, virtual screening against the β-haematin crystal was performed in two steps. Initially, the search for possible crystal growth inhibitors by docking was performed with AutoDock Vina assembled in the PyRx Virtual Screening Tool^16^. This was followed by *in silico* toxicological and drug-likeness filtering using DataWarrior (Fig. 1)^17^.

Once docking was completed, a binding affinity of −10 kcal/mol was chosen as the cut-off for the next step, and the top ranked 324 compounds (4.6%) were exported for toxicity prediction. Toxicity was predicted by calculating the risk of a compound having mutagenic, tumorigenic, reproductive or irritant effects^17^.

Oral bioavailability predictions were made using Lipinski’s rule of five (Ro5)^18^ excluding compounds with more than ten hydrogen-bond acceptors and five hydrogen-bond donors^19^. Compounds that scored negative in drug-likeness were then excluded. Ro5 represents a set of simple cheminformatics filters or drug-likeness filters that predict bioavailability of orally absorbed compounds. Drug candidates that conform to Ro5 tend to have good success rates during clinical trials and an enhanced probability of reaching the pharmaceutical market^19,20^.

The remaining compounds were visually inspected for favourable interactions such as π-π stacking, hydrogen-bonds and electrostatic interactions with the crystal surface. The test compounds were finally selected from the top ranked compounds sorted by their Vina binding affinity. Of these, a number were identified as interesting or as having many favourable interactions and fifteen compounds were purchased for further investigation (Table 1).

**Table 1:** Virtual screening results showing the top-ranked compounds that were purchased for experimental analysis.

**Compound**	**Structure**	**Vina Binding Affinity (kcal/mol)**	**Drug-Likeness**
**1** (Zinc000011972569)		−14.2	5.65
**2** (Zinc000012313974)		−14.1	7.44
**3** (Zinc000008583067)		−14	6.63
**4** (Zinc000003644958)		−14	6.05
**5** (Zinc000032960909)		−13.8	1.75
**6** (Zinc000043931987)		−13.7	2.95
**7** (Zinc000032960833)		−13.6	1.79
**8** (Zinc000012556326)		−13.2	3.78
**9** (Zinc000032960924)		−13	0.09
**10** (Zinc000006782501)		−12.8	6.68
**11** (Zinc000040451180)		−12.7	6.92
**12** (Zinc000006782511)		−12.6	6.68
**13** (Zinc000032960619)		−12.6	0.126
**14** (Zinc000032960813)		−12.6	1.8
**15** (Zinc000031938586)		−12.5	8.58

**Figure 2:**
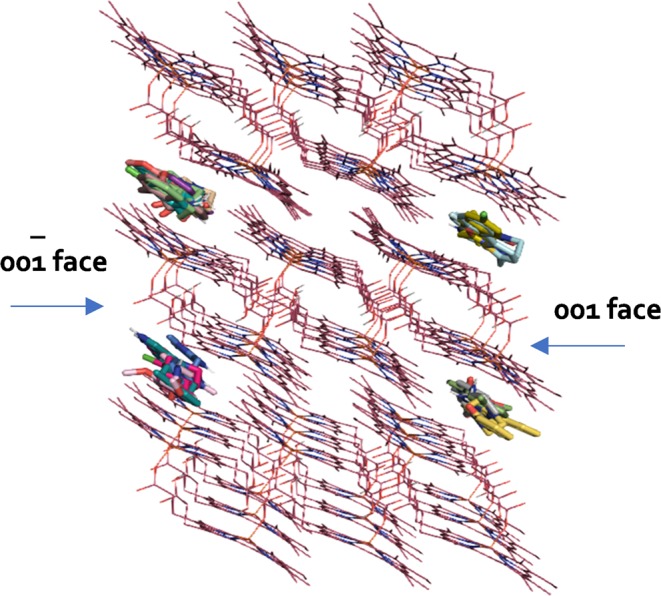
3D structure of the β-haematin crystal showing the top ranked compounds found by virtual screening exhibiting a preference for docking with the 001 and 00 $\bar{1}$ faces.

Based on the crystal structure of β-haematin and the low concentration of the drug CQ that is found in the blood stream, Pagola *et al*. inferred that the quinoline antimalarials may act through growth inhibition of haemozoin crystals by adsorption onto the actively growing faces or fastest growing faces resulting in a build-up of toxic haem and thus the death of the parasite^9^. A theoretical study of the β-haematin growth form proposed a noncovalent binding site for quinolines between porphyrin rings within crevices in the highly corrugated fastest-growing face [001]^5^. Analysing the size and morphology of crystals grown in presence of drugs like CQ and AQ, Vekilov and co-workers observed shorter and tapered average crystal shapes, indicating preferential inhibition of the axial and fastest growing faces, [001] and/or [011] in citrate buffer-saturated octanol^7^. Our docking simulations made use of a search space covering the whole surface of the β-haematin crystal allowing the compounds to dock with any preferential face and interestingly the top ranked compounds also showed a preference for the crevices on the corrugated fastest growing 001 face (Fig. 2). It should be noted, however, that while the strongest interaction is predicted to be with the [001] face, this does not imply that no interaction occurs with other faces. Indeed, in a recent investigation of benzimidazole haemozoin inhibitors we found that docking to both the [001] and [011] faces needed to be considered to explain observed activity^21^.

### Experimental tests

Compounds were evaluated for their inhibition of β-haematin formation in an NP-40 detergent mediated assay using pyridine to detect unreacted haematin^22,23^. In addition, parasite growth inhibition was tested against the chloroquine- and pyrimethamine-resistant *P*. *falciparum* K1 strain cultured *in vitro* (Table 2)^24,25^.

**Table 2:** Inhibition of β-haematin formation, antiparasitic activity (K1 strain of *P*. *falciparum*), cytotoxicity in a Chinese hamster ovarian (CHO) mammalian cell-line, and selectivity index (SI) of the purchased compounds.

**COMPOUND**	**β-haematin IC**_**50**_ **(μM)**	**K1 IC**_**50**_ **(μM)**^**a**^	**CHO IC**_**50**_ **(µM)**	**SI**^**b**^
**1** (Zinc000011972569)	305 ± 37	NA	—	
**2** (Zinc000012313974)	339 ± 7	5.81 ± 0.0021	—	
**3** (Zinc000008583067)	>1,000	NA	—	
**4** (Zinc000003644958)	288 ± 13	NA	—	
**5** (Zinc000032960909)	>1,000	NA	—	
**6** (Zinc000043931987)	64 ± 3	3.0 ± 0.9	18.77 ± 0.0005	6.34
**7** (Zinc000032960833)	>1,000	NA	—	
**8** (Zinc000012556326)	139 ± 22	18.8 ± 0.3	—	
**9** (Zinc000032960924)	66.3 ± 0.2	4.9 ± 1.5	—	
**10** (Zinc000006782501)	394 ± 79	NA	—	
**11** (Zinc000040451180)	212 ± 57	2.3 ± 0.1	13,889 ± 6	6,012.6
**12** (Zinc000006782511)	605 ± 64	10.9 ± 0.6	—	
**13** (Zinc000032960619)	313 ± 25	19 ± 2	—	
**14** (Zinc000032960813)	>1,000	20.7 ± 0.2	—	
**15** (Zinc000031938586)	563 ± 65	12.0 ± 0.3	—	
Chloroquine (CQ)	22 ± 3	0.17 ± 0.03	—	

^a^NA = not active at tested concentration of 10 µg/mL.

^b^SI = Selectivity index = CHO IC_50_/K1 IC_50_.

A notable highlight of the findings in Table 2 is that 11 out of 15 compounds inhibited β-haematin formation, revealing a remarkable hit rate of 73%. Compounds **6** and **9** showed the best activity, with a β-haematin growth inhibition IC_50_ below 100 μM, similar to that of clinical hemozoin inhibiting antimalarials (22.0 μM for chloroquine and 52.0 μM for quinine)^26,27^.

The benzimidazole moiety was found in five out of the fifteen compounds (compounds **5**, **7**, **9**, **13** and **14**). Benzimidazoles are a recognised chemical class that have been previously shown to inhibit the growth of *Plasmodium* parasites^28,29^. Singh *et al*. have shown β-haematin inhibitory activities for benzimidazole compounds^30^. In an earlier work, our research group found the benzimidazole ring was the foremost structure in a Bayesian model of haemozoin-inhibiting compounds active against *P*. *falciparum*. It was found that 103 of 155 compounds with this fingerprint were active in the β-haematin inhibition assay while 194 of 194 compounds were active in the parasite growth inhibition activity assay^31^. That study led to the synthesis of benzimidazole analogues, 83% of which were found to inhibit β-haematin formation and 50% inhibited parasite growth^21^. Although **9** showed one of the best β-haematin inhibitory activities, curiously, three out the five benzimidazole compounds identified in this series (compound **5**, **7**, and **14**) were β-haematin and parasite inactive (with compound **14** weakly active against *P*. *falciparum*). Notably, all these inactive benzimidazoles have an N-methyl group on the imidazole ring.

Analysing the parasite growth inhibition data, nine compounds showed activity, giving a significant 60% hit rate. Compounds **6** and **11** showed the best inhibitory activity and interestingly both have a benzoxazole scaffold. Even though compound **11** did not exhibit the best activity against β-haematin formation, it was chosen together with compound **6** for further investigation. Chibale and co-workers have reported the synthesis and evaluation of the antiplasmodial activity of benzothiazole, benzimidazole, benzoxazole and pyridine analogues of amodiaquine. The benzoxazole compounds showed an excellent activity against *P*. *falciparum*^32^. Nonetheless, there is currently little published data on the benzoxazole moiety with regards to antimalarial activity and/or β-haematin inhibition activity, although it was identified as a hit in a β-haematin HTS performed by Sandlin *et al*. showing parasite growth inhibitory activity as well^33^. Thus, this scaffold is of potential interest for further development.

### Cytotoxicity and haem fractionation assay

The compounds most active against the parasite (**6** and **11**) were then tested for cytotoxicity against mammalian cells (Chinese hamster ovarian, CHO, cells). The selectivity index is a measure of selectivity of a compound towards the desired target or cell and in this case is the ratio of the CHO IC_50_ to parasite IC_50_. As shown in Table 2, compound **11** had a much better SI than **6**, which showed rather poor selectivity. This is an indication that the activity of **6** might be attributed its toxicity towards cells. In the case of **11**, the activity indicates excellent selectivity of the compound towards the parasite.

**Figure 3:**
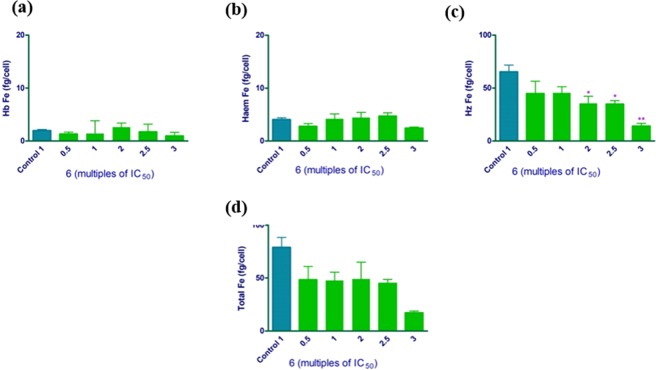
Levels of intraparasitic haemoglobin (**a**), freely exchangeable haem (**b**), haemozoin (**c**) and total intraparasitic haem (**d**) in isolated *P*. *falciparum* trophozoites following 32 h incubation with compound **6** at multiples of its IC_50._ Control: untreated cells. Levels are expressed as fg of haem Fe per cell. Statistical significance was calculated using a two-tailed t-test (error bars showing 95% CI) and is expressed relative to the control using asterisks.

To ascertain whether the ability of these compounds to inhibit β-haematin translates into haemozoin inhibition in the parasite, we conducted a cellular haem fractionation assay^34^. This assay measures exchangeable haem, haemozoin and undigested haemoglobin in cultured *P*. *falciparum* cells. As can be seen from Fig. 3, there is no change in the amount of freely exchangeable haem (represented as haem Fe in fg/cell), which implies that an increase in the concentration of **6** results in no increase in free haem within the parasite. This suggests that the mechanism of action of **6** is not *via* haemozoin inhibition. Indeed, similar observations have previously been made with atovaquone which is not a haemozoin inhibitor^34^. Further investigation would be required to clarify its mechanism of action.

**Figure 4:**
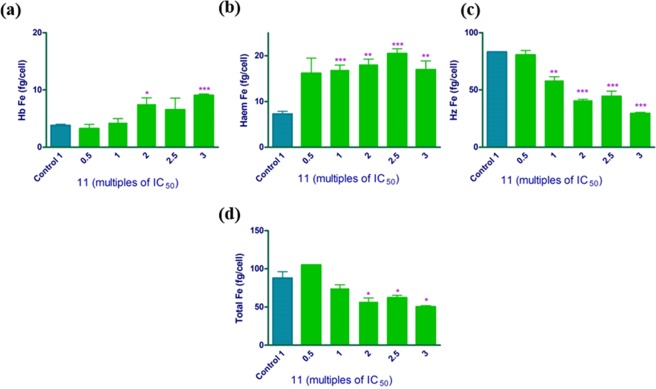
Intraparasitic haemoglobin (**a**), freely exchangeable haem (**b**), haemozoin (**c**) and total intraparasitic haem (**d**) in isolated *P*. *falciparum* trophozoites following 32 h incubation with compound **11** at multiples of its IC_50._ Control: untreated cells, with haem expressed in fg Fe/cell. Statistical significance was calculated using a two-tailed t-test (error bars showing 95% CI) and is expressed relative to the control using asterisks.

Compound **11**, on the other hand, was found to decrease haemozoin in a dose dependent manner (Fig. 4). There was a corresponding significant dose-dependent increase in freely exchangeable haem, confirmed by an unpaired t-test relative to control. This compound shows a very similar haem fractionation profile to that observed for CQ in previous studies^4,34^. The statistically significant dose-dependent increase in haemoglobin is in agreement with these earlier studies that exhibited an increase in undigested haemoglobin in parasites treated with CQ which only became apparent at concentrations well above the IC_50_. The dose response curve for exchangeable haem is tightly correlated with parasite growth inhibition, with free haem appearing before undigested Hb, strongly suggesting that the increase in undigested haemoglobin follows the effects of free haem on the parasite as previously argued^4^. Overall, the evidence strongly indicates that compound **11** acts primarily by inhibiting cellular haemozoin formation. Although the detailed mechanism of how haem then kills the parasite is still a subject of investigation, this result is aligned with the hypothesis that an increase in free haem is ultimately responsible for parasite killing^34^. In the case of CQ it has been argued the low pH of the digestive vacuole results in the uncharged protonation state of Fe(III)haem predominating. This species is hydrophobic and is known to interact with lipids, possibly allowing soluble haem to diffuse out of the digestive vacuole where it would be deprotonated in the parasite cytoplasm, forming anionic species. In the case of CQ we reported evidence that this redistributed haem is associated with membranous structures in the cytoplasm, possibly the endoplasmic reticulum. It may be that destabilization of membranes in the cell, possibly through formation of reactive oxygen species, leads to parasite death. This may involve multiple effects, which include disruption of ion homeostasis, membrane trafficking, and small molecule transport^4^. In the case of CQ, a fairly strong complex can form with Fe(III)haem and it may be that haem is mobilised as a complex. This may also be the case with **11**, but its relatively low solubility in aqueous solution precluded measurement of its association with Fe(III)haem.

One aspect of haemozoin inhibiting drugs is that they can be pH trapped in the acidic DV of the parasite and this has been correlated before with the IC_50_ of chloroquine and analogues^35^. The benzoxazole **11** is much less basic than chloroquine and can only be protonated around pH 5 on the piperidine ring which contains the most basic nitrogen. It can be expected that it will accumulate in the DV to a much smaller extent than clinical haemozoin inhibitors. Nonetheless, **11** showed a higher drug-likeness score than compound **6** (6.92 against 2.95, Table 1), predicting **11** as a good candidate for further development.

A number of HTS studies based on inhibition of β-haematin formation have been reported in the scientific literature^33,36–39^. Hirayama and co-workers performed a HTS on 9,600 compounds assigned randomly from the chemical library of The Drug Discovery Initiative, Tokyo University. In total, 394 β-haematin inhibitors were identified showing a hit rate of 4.1%^40^. Sandlin *et al*. screened 144,330 compounds for the identification of inhibitors of β-haematin crystallization, resulting in 530 hits: a hit rate of 0.37%^33^. Our study showed an experimental β-haematin inhibition hit rate after VS of 73%, exhibiting a clear enrichment (17- to 200-fold) over random screening, demonstrating that VS can be a useful and cost-effective approach in the search for new haemozoin inhibitors, saving time and money.

Recently, ligand based virtual screening applying Bayesian classifiers based on Bayes’ theorem has been used to build *in silico* activity models trained with HTS data for predicting β-haematin inhibition and *in vitro* antimalarial activity^21,31,40,41^. However, to our knowledge, this is the first study applying SBVS for identification of new actives against β-haematin.

It is predicted that the malaria burden will continue to be high for decades. This, together with the loss of antimalarials to resistance, shows that new alternatives will be needed for the coming years^42,43^. VS may have a role producing a significant enrichment of potential inhibitors, maximising efficiency and reducing costs in the discovery of new antimalarial drugs.

The primary purpose of this pilot study was to explore the feasibility of using SBVS for the discovery of new hemozoin inhibitors with activity against *P*. *falciparum*. This has been clearly demonstrated. The findings also suggest new avenues of research that could be pursued in future projects. These include experimental investigation of mechanisms of inhibition of β-haematin formation, modes of parasite killing and medicinal chemistry investigations based on compound **11** to improve activity and solubility as well as to study the pharmacological properties of this class of compound.

## Methods

### Data collection and preparation

To identify new β-haematin formation inhibitors, the ZINC15 database was chosen. ZINC15 is a database with public access established to allow ready access to compounds for virtual screening. ZINC15 has more than 210 million purchasable lead-like 3D compounds; all molecules are available in a biologically relevant, ready-to-dock format^15^. Of the 3D structures available, a subset was selected that fitted a specific set of criteria. In this case only compounds that had clean reactivity; were in stock; with neutral charges were considered. Of these, compounds below a LogP of 5 and molecular weight of 425 were selected, resulting in a final number of 7,070 compounds (fetched: June/2017).

The compounds were imported into OpenBabel within the Python Prescription Virtual Screening Tool (PyRx)^16^ and subjected to energy minimisation. The energy minimisation was performed with the Universal Force Field (UFF) using the conjugate gradient algorithm. The total number of steps was set to 200 and number of steps for update set to 1. In addition, the minimisation was set to stop at an energy difference of less than 0.1 kcal/mol.

### Virtual screening

Structure- based virtual screening applying docking simulations was performed using the AutoDock Vina^44^ tool compiled in PyRx^16^. Here, the structure of the β-haematin crystal previously published was used as the macromolecule (receptor)^9^. The search space encompassed the whole of the modelled crystal (made up of 27 unit cells) with the following dimensions in Å: centre (x, y, z) = (18, 20, -14), dimensions (x, y, z) = (41, 53, 36). The docking simulation was then run at an exhaustiveness of 8 and set to only output the lowest energy pose.

After the docking was complete, the top ranked compounds were exported to Osiris DataWarrior which was used to eliminate compounds with predicted toxicity and/or poor bioavailability^17^. The toxicity is predicted by comparison to a precompiled fragment library derived from the RTECS (Registry of Toxic Effects of Chemical Substances) database^17^. In addition to this, a drug-likeness calculation was performed, which is based on a library of about 5,300 substructure fragments and their associated drug-likeness scores. This library was prepared by fragmenting 3,300 commercial drugs as well as 15,000 commercial non-drug-like Fluka compounds^17^. Finally, the docked poses with β-haematin were imported into PyMOL Molecular Graphics System to be visually inspected^45^.

### Compounds

Predicted inhibitors of β-haematin formation were purchased from Aurora (**1** and **2**), ChemDiv (**3–5**, **7**, **9–14**) and Enamine (**6**, **8**, **15**).

### Detergent mediated assay for β-hematin inhibition

The assay for measuring inhibition of β-haematin formation was based on that described by Carter *et al*.^22^. Compound solutions were made up to 20 mM in DMSO, the reference standard CQ was prepared in distilled water; and 20 µL of each compound was delivered in duplicate to the wells in column 12 of a 96-well plate. Distilled water (140 µL) and the detergent NP-40 (307.3 μM, 40 µL) was also added to each of these wells. A solution consisting of water/NP-40 solution (307.3 μM)/DMSO in a ratio 7:2:1 (v/v) was prepared and 100 µL was then added to the wells in columns 1–11 of the plate. Serial dilution of each compound (100 µL) from column 12 to column 2 was then performed leaving column 1 as a blank. A haematin stock solution (25 mM) was prepared from haemin by sonicating in DMSO for 1 min followed by introduction of 178.8 µL of this solution to 1 M acetate buffer (20 mL, pH 4.8). 100 µL of this homogenous suspension was then added to all wells. The plate was covered and incubated for 5 h at 37 °C in an incubator. The pyridine-ferrochrome method developed by Ncokazi and Egan was used to perform the analysis^23^. This method is based on the principle that aqueous pyridine forms a soluble low spin complex with the Fe(III) centre in haematin but not haemozoin, and since the absorbance obeys Beer’s law, it allows for quantification of haem concentration in solution^23^. A solution consisting of 50% (v/v) pyridine, 30% (v/v) H_2_O, 20% (v/v) acetone and 10% (v/v) 2 M HEPES buffer (pH 7.4) was prepared and 32 μL added to each well. Acetone (60 μL) was added to assist haematin dispersion. The UV-vis absorbance of the plate was read at 405 nm on a Thermo Scientific Multiskan GO plate reader. The sigmoidal dose-response curves were plotted using GraphPad Prism version 6 (GraphPad Software Inc., La Jolla, CA, USA) to calculate the IC_50_ of each compound.

### *Plasmodium* lactate dehydrogenase (pLDH) assay (*in vitro* anti-plasmodial assay)

Culturing of parasites followed the earlier method by Trager and Jensen^24^ and the LDH assay was based on that of Makler *et al*.^25^. The K1 strain of *P*. *falciparum* (chloroquine and pyrimethamine resistant) was used to test *in vitro* antimalarial activity. This assay was used to evaluate the predicted activity of the compounds obtained by virtual screening. It is based on detecting the presence of *P*. *falciparum* lactate dehydrogenase activity after 48 h incubation in a 96-well plate. The IC_50_ values were obtained using non-linear dose-response curve fitting analysis via GraphPad Prism v 5.0.0 software (GraphPad Software Inc., La Jolla, CA, USA).

### Cytotoxicity

Selected compounds were tested for *in vitro* cytotoxicity against a Chinese Hamster Ovarian (CHO) mammalian cell-line, using the 3-(4,5-dimethylthiazol-2-yl)-2,5-diphenyltetrazoliumbromide (MTT) assay. The CHO cells were seeded in a 96-well plate (3,000 cells per well) and then incubated at 37 °C under 5% CO_2_ for 24 h. Stock solutions of each compound (final DMSO concentration of 0.2%) were then added to the plates with concentrations ranging from 100 µg/mL to 0.001 µg/mL (six 10-fold dilutions) and the plates were incubated for 48 h. Cell viability was then measured by the MTT assay that is based on the reduction of the tetrazolium salt (MTT), by live cells during proliferation^46^. Absorbance was read at 595 nm, and the IC_50_ values calculated using GraphPad Prism v 5.0.0 software (GraphPad Software Inc., La Jolla, CA, USA).

### Haem fractionation assay

Target validation for compound **6** and **11** was carried out via a cell fractionation assay, optimized to a multi-well colorimetric assay for determining haem species in *P*. *falciparum* as described by Combrinck *et al*.^34^. The cellular fractionation allows for direct quantification of the three major haem species in isolated trophozoites namely; haemoglobin (Hb), freely exchangeable haem, and haemozoin. Target validation is evaluated by measuring the increase in freely exchangeable haem and the decrease in haemozoin formation.

### Statistical analysis

A two-tailed t-test (95% CI) was used for determination of statistical significance of differences in measurements relative to controls, and is displayed using asterisks on graphs (*P < 0.05; **P < 0.01; ***P < 0.001). The data represents a minimum of three repeats with standard deviations calculated for each of the average results. All the analysis was done using GraphPad Prism version 5.0.0 software.

## Data Availability

All data produced or investigated during this research are included in this published article. Requests for additional materials should be addressed to the corresponding author.
